# High Expression of Tomm34 and Its Correlations With Clinicopathology in Oral Squamous Cell Carcinoma

**DOI:** 10.3389/pore.2021.641042

**Published:** 2021-04-16

**Authors:** Min Cai, Rukeng Tan, Yunyi Huang, Xuanyi Chen, Qingci Kong, Kaixin Guo, Meng Xu

**Affiliations:** Hospital of Stomatology, Guanghua School of Stomatology, Sun Yat-sen University, Guangdong Provincial Key Laboratory of Stomatology, Guangzhou, China

**Keywords:** oral squamous cell carcinoma, Tomm34, Immunohistochemistry, clinicopathology, bioinformatics

## Abstract

Tomm34, as a member of the outer mitochondrial membrane proteins, is evenly distributed between the cytoplasm and the outer mitochondrial membrane. It is up-regulated in a variety of tumors and correlates with poor prognosis. This study aimed to investigate expression of Tomm34 and its correlations with clinicopathology in oral squamous cell carcinoma (OSCC). Oncomine database and UALCAN database were utilized to predict the expression and prognosis values of Tomm34 in head and neck squamous cell carcinoma (HNSCC). By immunohistochemistry, a retrospective study was performed to verify the bioinformatics results to evaluate the Tomm34 expression and clinicopathological variables in both HPV-positive OSCC and HPV-negative OSCC. Immunohistochemistry of our cohort revealed that 48 cases fulfilled the Tomm34 high expression judgment criteria, and the overall positive rate was 60% (48/80), and 27 cases fulfilled the p16 expression judgment criteria (33.75%, 27/80). The high expression of Tomm34 was closely related with the TNM classification of OSCC (*p* < 0.01) and tumor size (*p* < 0.01) both in HPV-negative OSCC and HPV-positive OSCC, while related with lymph node metastasis (*p* = 0.001) in HPV-negative OSCC and drinking history (*p* = 0.044) in HPV-positive OSCC. In addition, the Kaplan-Meier curves indicated that higher level of Tomm34 was correlated with poorer overall survival (OS) and disease-free survival (DFS) in HPV-negative OSCC (OS, *p* = 0.046; DFS, *p* = 0.020) but not in HPV-positive OSCC (OS, *p* = 0.824; DFS, *p* = 0.782). In conclusion, Tomm34 is highly expressed in OSCC and may be a useful factor to provide prognostic information, especially in HPV-negative OSCC group.

## Introduction

Head and neck squamous cell carcinoma (HNSCC), accounts for approximately 80% of head and neck tumors, is highly aggressive with frequent local recurrences and lymph node metastases [1]. Oral cavity, oropharynx, larynx are the most common sites of HNSCC [2]. The routine therapy for oral squamous cell carcinoma (OSCC) patients is radical surgical resection combined with chemotherapy and radiotherapy, whereas the outcome is unsatisfactory. About half of patients with advanced cancer die within 5 years [3, 4]. Early diagnosis of OSCC is important in patient treatment and prognostic evaluation. Therefore, it is worthwhile to reveal novel diagnostic and prognostic biomarkers for OSCC.

Recently, the role of high-risk human papilloma virus (HPV) infection in HNSCC has been well-confirmed [5]. The HPV-associated oropharyngeal carcinoma (HPV-OPC) has been considered as a distinct entity according to the WHO classification (4^th^ edition) [6–8], in addition, OSCC has been validated to harbor the virus as well. It is widely demonstrated that p16 immunohistochemistry can be utilized as a surrogate marker to classify the HPV status in OPC and OSCC [5, 9]. HPV-positive OPC exhibited a more favorable prognosis than HPV-negative OPC, however, the mechanism of better prognosis of HPV-positive OSCC compared with HPV-negative OSCC has not been fully understood yet.

Tomm34 (34-kDa translocase of the outer mitochondrial membrane), a member of the TOMM family, locates on chromosome 20q13.12 and contains 18,357 bases and 6 TPR repeats [10, 11]. It is evenly distributed between the cytoplasm and the outer mitochondrial membrane and participates in protein transport [12]. Tomm34 can interact with Hsp70 and Hsp90 to form Hsp70-Tomm34-Hsp90 complex, which acts as a co-chaperon of Hsp70 and Hsp90 to regulate its function [13, 14]. Recent studies showed high levels of Tomm34 expressed in colorectal cancer [15], hepatocellular carcinoma [16], bladder cancer [17], breast cancer [18, 19] epithelial ovarian cancer [20] and colon cancer [21] compared to their normal tissue counterparts. However, the expression of Tomm34 in oral squamous cell carcinoma primary tumor has not been reported and its role in OSCC is still unclear.

In the present study, we aimed to investigate the expression of Tomm34 in OSCC. Oncomine database and UALCAN database were utilized to predict the expression and prognosis values of Tomm34 in HNSCC. By immunohistochemistry, a retrospective study was performed to verify the bioinformatics results to evaluate Tomm34 expression and clinicopathological variables.

## Materials and Methods

### Tomm34 Expression in HNSCC Analyzed by Oncomine Database

Oncomine is an online data-mining platform which contains various microarray expression data. The data of Tomm34 gene expression in HNSCC was extracted from Oncomine database. The search conditions were as follows: 1) Gene: Tomm34; 2) Analysis Type: Cancer vs. Normal Analysis; 3) Cancer Type: Head and Neck Squamous Cell Carcinoma; 4) Analysis parameters were *p*-value < 1E-4, fold change >2, gene rank = 10%.

### Kaplan-Meier Survival Analysis of the Tomm34 Analyzed by UALCAN

UALCAN is a comprehensive, user-friendly, and interactive web resource for analyzing cancer OMICS data [22]. Transcripts per million (TPM) values were used to measure the expression level of Tomm34, categorized into high expression group (TPM values are above the upper quartile) and low/medium expression group (TPM values are below the upper quartile) [22]. We compared the expression of Tomm34 in normal tissues and HNSCC, and analyzed the correlation of Tomm34 with clinicopathological variables such as tumor stage, tumor classification, race and weight and other indicators. Kaplan-Meier curves of Tomm34 expression and survival time were also evaluated.

### Primary OSCC Samples

80 archival formalin-fixed paraffin embedded (FFPE) primary OSCC biopsies were recruited from the tissue bank of Department of Oral Pathology, Hospital of Stomatology, Guanghua School of Stomatology, Sun Yat-sen University, Guangzhou, China for immunohistochemical (IHC) staining study. All of the specimens were histologically evaluated as squamous cell carcinoma which originated from oral cavity and oropharynx including tongue, buccal mucosa, gingiva, floor of mouth, hard palate, oropharynx and soft palate. Clinicopathological informations including gender, age, tumor size, lymph node metastasis, pathology differentiation, TNM status, disease-free survival (DFS) and overall survival (OS) were analyzed. The study protocol was approved by Clinical Research Ethics Committee of Hospital of Stomatology, Guanghua School of Stomatology, Sun Yat-sen University.

### Immunohistochemistry

FFPE OSCC specimens were cut into 4 μm thick sections, de-paraffinized in xylene, and rehydrated in serial dilutions of ethanol. Endogenous peroxidase activities were subsequently blocked by a 10-min treatment with 3% H_2_O_2_ solution. Antigen retrieval was performed in EDTA (pH 9.0) for 2 min at 10.0 MPa. Following incubation in 5% BSA for 10 min to block endogenous biotin activity, sections were incubated with the primary antibody (Tomm34, 1:100, Proteintech Inc., United States; p16, 1:200, Abcam, United States) at 4°C overnight. The HRP-labeled secondary antibody was applied to the sections and incubated at 37°C for 40 min. For color development, sections were stained 3,3′-diamino-benzidine (DAB) substrate and hematoxylin. Human colon carcinoma tissue was utilized as the positive control, and samples which were incubated with PBS instead of primary antibody served as the negative controls.

The extent and intensity of staining were evaluated and scored by two independent pathologists. Twenty microscopic high-power fields of tumor tissues were randomly selected for assessment. A numeric intensity score of 0–4 was assigned to each case based on the percentage of positive neoplasm cells (0, negative, <10% positive cells staining; 1+, 10–25% positive cells with weak staining; 2+, 25–50% positive cells with moderate staining; 3+, 51–70% positive cells with strong staining and 4+, >70% positive cells with strong staining). Staining was dichotomized into low expression and high expression by score. Score 0–2 was considered as low expression of Tomm34, and score 3–4 was considered as high expression of Tomm34. Meanwhile, >70% positive cells with strong and diffuse nuclei staining (with or without cytoplasmic staining) (score 4) was considered as positive expression of p16 [9].

### Statistical Analyses

Data was analyzed by IBM SPSS statistical software V22 (IBM Corporation, United States). The χ2 test was utilized to assess the associations between the expression of Tomm34 or p16 and clinical characteristics of patients. Survival estimates were analyzed by Kaplan-Meier method with significance determined by the log rank test. A *p* value less than 0.05 (two-sided) was considered significant.

## Results

### Tomm34 Expression in Different Tumors by Oncomine Database

**FIGURE 1:**
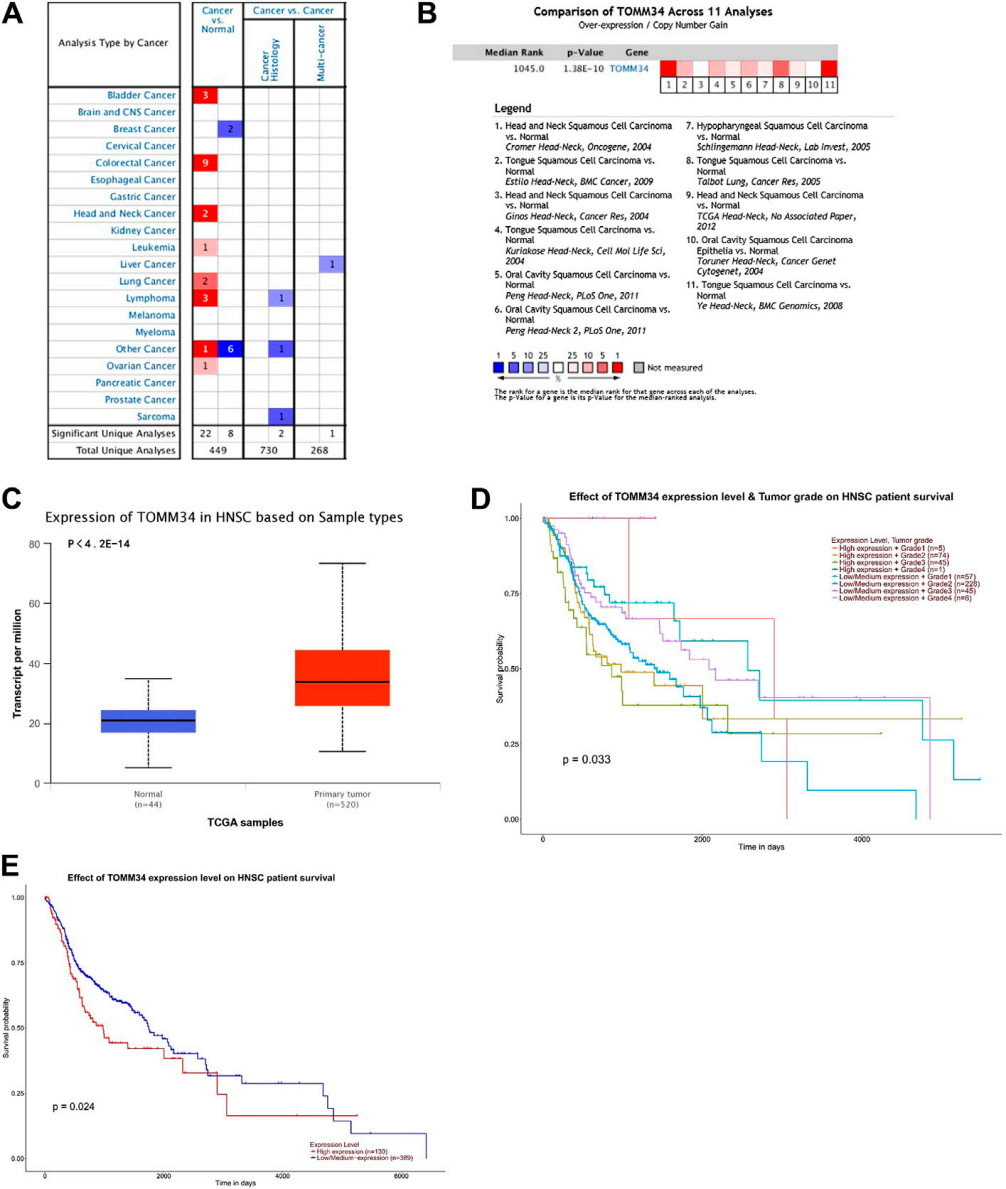
**(A)** Tomm34 expression levels in human cancer tissues. **(B)** Comparison of Tomm34 across 11 analyses in HNSCC. **(C)** Expression of Tomm34 based on HNSCC sample types. **(D)** Tomm34 expression varies in HNSCC with different grades (The categories of tumor grade: Grade1, well differentiated; Grade2, moderately differentiated; Grade3, poorly differentiated; Grade4, undifferentiated [22]). **(E)** Kaplan-Meier survival tests comparing in the high and low/medium expression of Tomm34 in HNSCC.

A total of 449 cases of different tumors of Tomm34 gene research results were collected in the Oncomine database. 30 research results showed statistical differences, where 22 of 30 cases reported overexpression of Tomm34 in tumor tissues and 8 cases reported lower expression in tumor tissues (Figure 1A).

### Tomm34 Expression in HNSCC by Oncomine Database

Tomm34 gene ranked in 1,045 position among the total expressed genes of HNSCC(*p* = 1.38 E −10) (Figure 1B). Moreover, Tomm34 expression was significantly upregulated in the HNSCC tissues paired normal tissues (Figure 1C).

### Tomm34 Expression and Clinicopathological Correlations in HNSCC Analyzed via UALCAN

For further study, the expression of Tomm34 in HNSCC was investigated in subgroups via UALCAN analysis. Tomm34 expression varied in HNSCC with different grades (Figure 1D). The Kaplan-Meier curves showed that higher level of Tomm34 correlated with poorer overall survival (OS) (Figure 1E).

### Tomm34 Expression and Its Correlations With Clinicopathology in Primary OSCC

**FIGURE 2:**
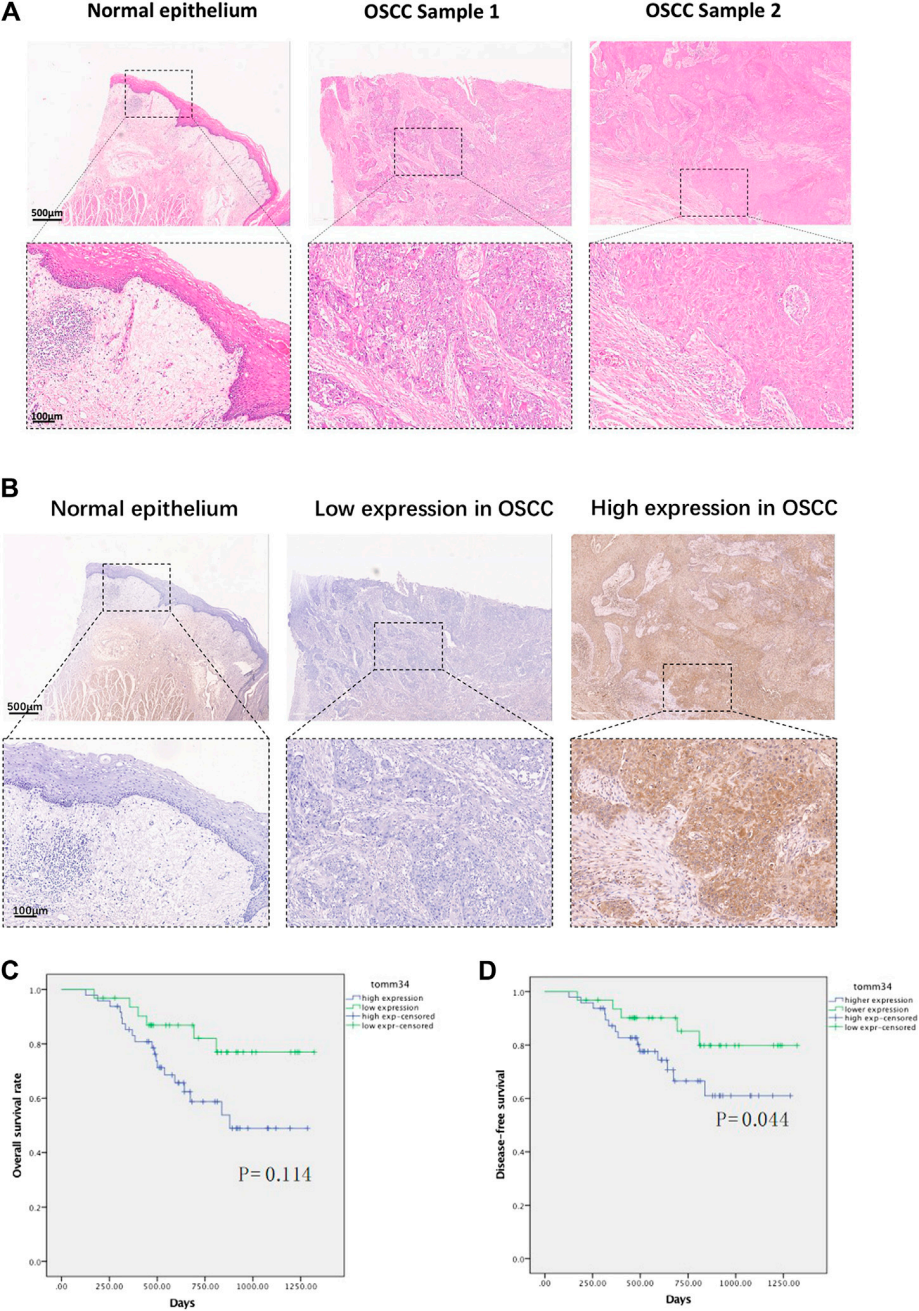
**(A)** Representative H&E staining images of normal epithelium and oral squamous cell carcinoma tissues. **(B)** Representative IHC images of Tomm34 expression in normal epithelium and oral squamous cell carcinoma tissues. **(C)** Survival curves of overall survival (OS) in OSCC cohort determined by Tomm34 expression level. **(D)** Survival curves of disease-free survival (DFS) in OSCC cohort determined byTomm34 expression level.

Tomm34 even staining was seen in the cytoplasm of tumor cells, nuclear expression was rarely seen (Figures 2A,B). Weak positive expression was seen in keratinocytes and some tumor stroma. In the cohort of 80 OSCC, 48 cases fulfilled the Tomm34 positive judgment criteria, and the overall positive rate was 60%.

The relationship between Tomm34 staining and clinicopathological factors were shown in Table 1. The expression of Tomm34 was closely related with the TNM clinical stage of OSCC (*p* < 0.001), tumor size (*p* < 0.001), lymph node metastasis (*p* = 0.001), drinking history (*p* = 0.019), but not with pathology differentiation, age, gender, smoking history and site (*p* > 0.05).

**TABLE 1:** Association between IHC expression of Tomm34 and clinicopathological parameters in the studied cohort (n = 80).

	Tomm34 expression		Significance	
	Negative	Positive	χ2	*p* Value
Age				
≤60 (years)	24	33	0.366	0.545
>60 (years)	8	15		
Gender				
Male	23	32	0.242	0.622
Female	9	16		
Tumor size				
T1/T2	30	24	16.752	<0.001
T3/T4	2	24		
Node category				
N^−^	27	23	0.668	0.001
N^+^	5	25		
Pathology differentiation				
Well	14	21	0.000	1.000
Moderate/poor	18	27		
TNM classification				
I/II	25	10	25.608	<0.001
III/IV	7	38		
Smoking				
Yes	8	18	1.368	0.242
No	24	30		
Alcohol (>5g/Day)				
Yes	2	13	5.470	0.019
No	30	35		
Site				
Buccal mucosa	2	9	4.705	0.453
Floor of mouth	3	6		
Gingiva	3	6		
Oropharynx and soft palate	2	2		
Hard palate	1	3		
Tongue	21	22		

To investigate the effects of Tomm34 on patient survival in more detail, we next assessed the relationship between the Tomm34 expression level and patient overall survival by Kaplan-Meier survival analysis. The overall survival was extremely down-regulated in the Tomm34-positive expression group compared with the Tomm34-negative expression group. The Kaplan-Meier curves indicated that higher level of Tomm34 correlated with poorer overall survival (OS, *p* = 0.114) and disease-free survival (DFS, *p* = 0.044) (Figures 2C,D).

### p16 Expression and Its Correlations With Clinicopathology in OSCC Cohort

**FIGURE 3:**
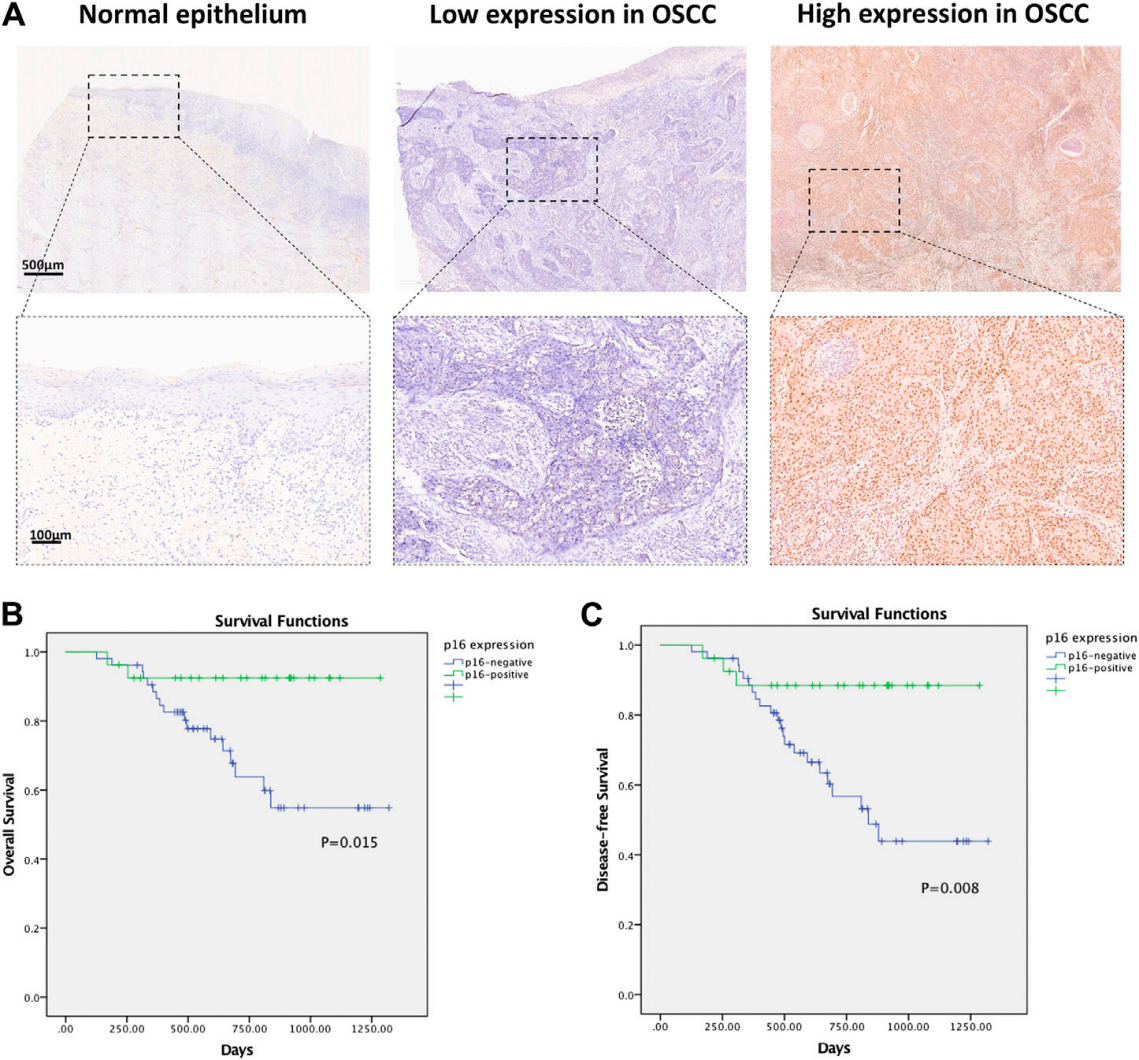
**(A)** Representative IHC images of p16 expression in normal epithelium and oral squamous cell carcinoma tissues. **(B)** Survival curves of overall survival (OS) in OSCC cohort determined by p16 status. **(C)** Survival curves of disease-free survival (DFS) in OSCC cohort determined by p16 status.

To separate the HPV-related cases in OSCC, p16 immunohistochemistry was utilized in the cohort. The expression of p16 was observed in 33.75% of the primary OSCC tumors (27/80), distinguished by strong staining in >70% (scored 4) of tumor cells. p16 was homogeneously detected in the nuclei and cytoplasm of tumor cells (Figure 3A).

The relationship between the p16 expression and clinicopathological factors was shown in Table 2. The expression of p16 was strongly associated with earlier TNM classification by WHO (*p* = 0.046) and lymph node metastasis (*p* = 0.003), but not with tumor site, age, gender, tumor size, pathology differentiation, cigarette and drinking history.

**TABLE 2:** Association between IHC expression of p16 and clinicopathological parameters in the studied cohort (n = 80).

	p16 expression		Significance	
	Negative	Positive	χ2	*p* Value
Age				
≤60 (years)	36	21	0.848	0.357
＞60 (years)	17	6		
Gender				
Male	37	18	0.082	0.774
Female	16	9		
Tumor size				
T1/T2	35	19	0.153	0.696
T3/T4	18	8		
Node category				
N^−^	27	23	8.949	0.003
N^+^	26	4		
Pathology differentiation				
Well	24	11	0.150	0.699
Moderate/poor	29	16		
TNM classification				
I/II	19	16	3.983	0.046
III/IV	34	11		
Smoking				
Yes	14	12	2.650	0.104
No	39	15		
Alcohol (>5g/Day)				
Yes	9	6	0.323	0.570
No	44	21		
Site				
Buccal mucosa	11	0	10.758	0.056
Floor of mouth	5	4		
Gingiva	5	4		
Oropharynx and soft palate	2	2		
Hard palate	3	1		
Tongue	27	16		

The relationship between the p16 expression level and patient overall survival was further assessed by Kaplan-Meier survival analysis. The overall survival rate was markedly down-regulated in the p16-negative group compared with the p16-positive group. The Kaplan-Meier curves indicated that patients with p16-positive staining exhibited better OS (*p* = 0.015) and DFS (*p* = 0.008) (Figures 3B,C).

### Tomm34 Expression in HPV-Positive OSCC Group and HPV-Negative OSCC Group

HPV-related status of this OSCC cohort has been described by p16 staining. To further analyze the relationship of Tomm34 and HPV status, the expression of Tomm34 was reassessed in HPV-positive OSCC (n = 27) and HPV-negative OSCC (n = 53), respectively. As shown in Table 3, the higher expression of Tomm34 was consistently correlated with larger tumor size (*p* = 0.01 in HPV-positive OSCC and *p* = 0.004 in HPV-negative OSCC) and higher TNM classification (*p* = 0.008 in HPV-positive OSCC and *p* < 0.001 in HPV-negative OSCC). However, drinking history was only related with Tomm34-postive expression group in HPV-positive OSCC (*p* = 0.044), while positive node category was only related with Tomm34-postive expression in HPV-negative OSCC (*p* = 0.001).

**TABLE 3:** Association between IHC expression of Tomm34 and clinicopathological parameters separated by p16-staining status (n = 80).

	p16-positive OSCC (n = 27)				p16-negative OSCC (n = 53)			
	Tomm34 expression		Significance		Tomm34 expression		Significance	
	Negative	Positive	χ2	*p* Value	Negative	Positive	χ2	*p* Value
Age								
≤60 (years)	10	11	*0.024*	***0.877***	14	22	*0.064*	***0.801***
＞60 (years)	2	4			6	11		
Gender								
Male	8	10	*0.000*	***1.000***	15	22	*0.410*	***0.522***
Female	4	5			5	11		
Tumor size								
T1/T2	12	7	*6.717*	***0.010***	18	17	*8.224*	***0.004***
T3/T4	0	8			2	16		
Node category								
N^−^	11	12	*0.902*	***0.762***	16	11	*10.852*	***0.001***
N^+^	1	3			4	22		
Pathology differentiation								
Well	6	5	*0.232*	***0.630***	8	16	*0.362*	***0.547***
Moderate/poor	6	10			12	17		
TNM classification								
I/II	11	5	*6.497*	***0.008***	14	5	*16.290*	***<0.001***
III/IV	1	10			6	28		
Smoking								
Yes	4	8	*1.080*	***0.299***	4	10	*0.680*	***0.410***
No	8	7			16	23		
Alcohol								
Yes	0	6	*4.074*	***0.044***	2	7	*0.458*	***0.499***
No	12	9			18	26		
Site								
Buccal mucosa	0	0	*4.712*	***0.403***	2	9	*4.150*	***0.576***
Floor of mouth	1	3			2	3		
Gingiva	1	3			2	3		
Oropharynx and soft palate	2	0			0	2		
Hard palate	0	1			1	2		
Tongue	8	8			13	14		

**FIGURE 4:**
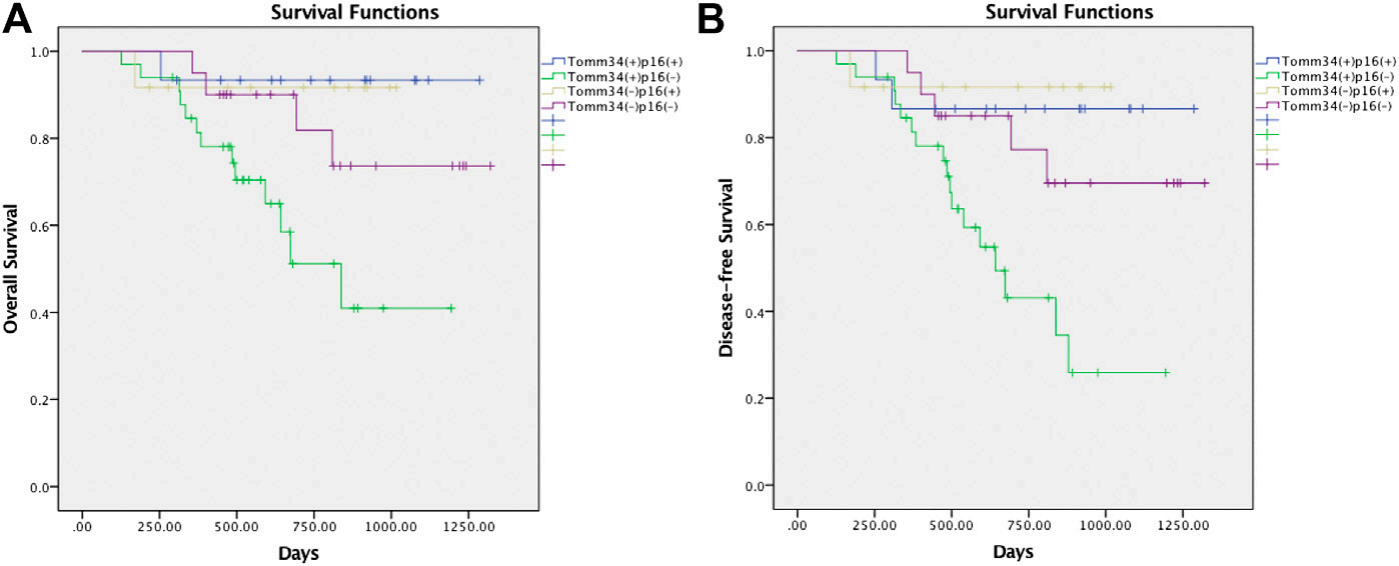
**(A)** Survival curves of overall survival (OS) in OSCC cohort determined by Tomm34 and p16 expression. **(B)** Survival curves of disease-free survival (DFS) in OSCC cohort determined by Tomm34 and p16 expression.

Furthermore, survival outcomes of four subgroups based on the expression of Tomm34 and p16 were examined (Figures 4A,B). Detailed pairwise comparisons of groups were shown in Table 4 (OS) and Table 5 (DFS). In HPV-positive OSCC subgroups, the survival curves of Tomm34 (+) p16 (+) group and Tomm34 (−) p16 (+) group exhibited rather little difference (OS, *p* = 0.824; DFS, *p* = 0.782). While in HPV-negative OSCC, statistically decreased OS (*p* = 0.046) and DFS (*p* = 0.020) rates were observed between Tomm34 (+) p16 (−) group and Tomm34 (−) p16 (−) group. In addition, Tomm34 (+) p16 (−) group also showed dramatically decreased survival rates compared with Tomm34 (+) p16 (+) group (OS, *p* = 0.014; DFS, *p* = 0.008) and Tomm34 (−) p16 (+) group (OS, *p* = 0.050; DFS, *p* = 0.015).

**TABLE 4:** Pairwise comparisons of Overall Survival Rates.

	Tomm34 (+), p16 (+)		Tomm34 (+), p16 (−)		Tomm34 (−), p16 (+)		Tomm34 (−), p16 (−)	
	Chi-square	Sig	Chi-square	Sig	Chi-square	Sig	Chi-square	Sig
Tomm34 (+),p16 (+)			6.007	***0.014***	0.050	***0.824***	0.979	**0.322**
Tomm34 (+), p16 (−)	6.007	***0.014***			3.835	***0.050***	3.980	**0.046**
Tomm34 (−), p16 (+)	0.050	***0.824***	3.835	***0.050***			0.522	**0.470**
Tomm34 (−), p16 (−)	0.979	***0.322***	3.980	***0.046***	0.522	***0.470***		

Pairwise comparisons were analyzed by Log Rank (Mantel-Cox).

**TABLE 5:** Pairwise comparisons of Disease-free Survival Rates.

	Tomm34 (+), p16 (+)		Tomm34 (+), p16 (−)		Tomm34 (−), p16 (+)		Tomm34 (−), p16 (−)	
	Chi-square	Sig	Chi-square	Sig	Chi-square	Sig	Chi-square	Sig
Tomm34 (+),p16 (+)			6.954	***0.008***	0.077	***0.782***	0.500	***0.480***
Tomm34 (+), p16 (−)	6.954	***0.008***			5.964	***0.015***	5.447	***0.020***
Tomm34 (−), p16 (+)	0.077	***0.782***	5.964	***0.015***			0.896	***0.344***
Tomm34 (−), p16 (−)	0.500	***0.480***	5.447	***0.020***	0.896	***0.344***		

Pairwise comparisons were analyzed by Log Rank (Mantel-Cox).

## Discussion

Mitochondria plays an important role in cell energy metabolism, senescence, apoptosis, and free radical formation [23]. Translocase of Outer Mitochondrial Membrane (TOMM) is one of the important proteins to maintain the normal function of mitochondria [24]. Tomm34, as a member of the outer mitochondrial membrane proteins, is evenly distributed between the cytoplasm and the outer mitochondrial membrane, and interacts with mitochondrial proteins in the cytoplasm to maintain the pre-protein in an unfolded and easy-to-introd state, and performs the function of a transport protein [10, 11]. Due to the instability of tumor genome and the lack of essential nutrients and oxygen, tumor cells need a lot of carbohydrates, proteins and other substances for growth and reproduction [25]. Tomm34 is considered to be increased as a component of compensatory adaptations to maintain normal rates of protein import in response to mitochondrial abnormalities in tumor. Our immunostaining data showed that Tomm34 stained predominantly and homogeneously in the cytoplasm rather than dot cluster coloring in mitochondria, suggesting it was involved in the transport of mitochondrial preproteins in an unfolded state prior to import, which was in agreement with others report.

Tomm34, a co-chaperone of Hsp70 and Hsp90, contains two TPR domains, N-terminal TPR1 and C-terminal TPR2 which binds to the highly conserved EEVD-COOH motif present in the C-terminal domain of Hsp70/Hsp90 [13, 14, 26, 27]. As increased chaperone activities are a universal feature of cancer [28], Hsp70-Tomm34-Hsp90 has been confirmed to be upregulated in a variety of cancers, including liver cancer [16], lung cancer [29], gastric cancer [30] and breast cancer [31], etc. Several studies have reported the immunoreactivity of Hsp70 and Hsp90 in OSCC, which were corelated with poor prognosis [32–35]. Tumor cells need a lot of carbohydrates, proteins and other substances for growth and proliferation which may up-regulate Hsp70 and Hsp90 proteins to synthesize more proteins [36]. Tomm34, as their co-chaperone, may also promote tumor growth by affecting the folding of Hsp70 and Hsp90. In the studied OSCC cohort, 33.75% of the primary tumors were p16 positive (27/80), which was in accordance with previous reports (10.0–38.1%) [5, 9]. Furthermore, our data showed that the expression of Tomm34 was observed in 60% of the primary OSCC (48/80). The higher expression level of Tomm34 was closely related with higher TNM classification of OSCC (*p* < 0.001) and larger tumor size (*p* < 0.001) in different HPV status. Lymph node metastasis (*p* = 0.001) was related with HPV-negative OSCC, while drinking history was related with HPV-positive OSCC (*p* = 0.044). Most notably, we have found that Tomm34 was associated with poor survival in HPV-negative OSCC rather than HPV-positive OSCC. These data are comparable to the findings that Tomm34 is a marker of poor outcome in bladder cancer and a predictor of distant metastasis in breast cancer.

Meanwhile, our data indicates that Tomm34 is a more sensitive prognosis marker for HPV-negative OSCC patients which is useful to evaluate patience’s risk of death. The mechanism for high level expression of Tomm34 is still unclear from our studies. As we known, TP53 mutation is a famous carcinogenetic factor of OSCC, which leads to the instability of the tumor genome [37]. Tomm34 expression levels indicates poor prognosis for p53-mutant epithelial ovarian cancer [20]. Our bioinformatic analysis indicated that higher expression of Tomm34 was detected in TP53-mutant OSCC compared with TP53-nonmutant OSCC and normal tissue (*p* < 0.001) (Supplementary Figure S1), and further research will be required to elucidate the regulation mechanisms. Recently, researchers have found Tomm34 is transcriptionally regulated by NRF-1 and NRF-2 under cancer stress and hyperactive condition during metabolic reprogramming [38, 39]. It has been shown that mice without Tomm34 expression can grow and reproduce normally, which has indicated that Tomm34 is not necessary for normal growth and development [40]. Tomm34 is not expressed in most important organs such as heart, liver, kidney and lung, which indicates that the side effects of using Tomm34 as an anti-cancer target may be minimal. Thus, clinical studies of vaccine therapy with an artificially synthesized cancer peptide based on the amino acid sequence of Tomm34 are ongoing. A phase I clinical trial of a peptide vaccine ring finger protein 43 (RNF43) and Tomm34 combined with uracil‐tegafur (UFT)/LV for patients with metastatic colorectal cancer has been done in Japan, including the safety and immunological responsiveness of this combination therapy [41]. Our data has provided evidence that Tomm34 is closely associated with clinicopathological parameters and may serve as a useful prognostic indicator and potential therapeutic target in OSCC, especially in HPV-negative OSCC.

## Conclusion

In summary, our study provides evidence that co-chaperone Tomm34 is frequently expressed in OSCC and may be a useful factor to provide prognostic information. Higher level of Tomm34 correlates with poorer overall survival and disease-free survival in HPV-negative OSCC but not in HPV-positive OSCC. Tomm34 may serve as a potential tumor biomarker for OSCC patients, especially in HPV-negative OSCC.

## Data Availability

The raw data supporting the conclusions of this article will be made available by the authors, without undue reservation.
