# Supplementary figures and images for: High Expression of Tomm34 and Its Correlations With Clinicopathology in Oral Squamous Cell Carcinoma
Source: Pathol Oncol Res. 2021 Apr 16;27:641042. doi: 10.3389/pore.2021.641042 (PMC8262227; doi:10.3389/pore.2021.641042)

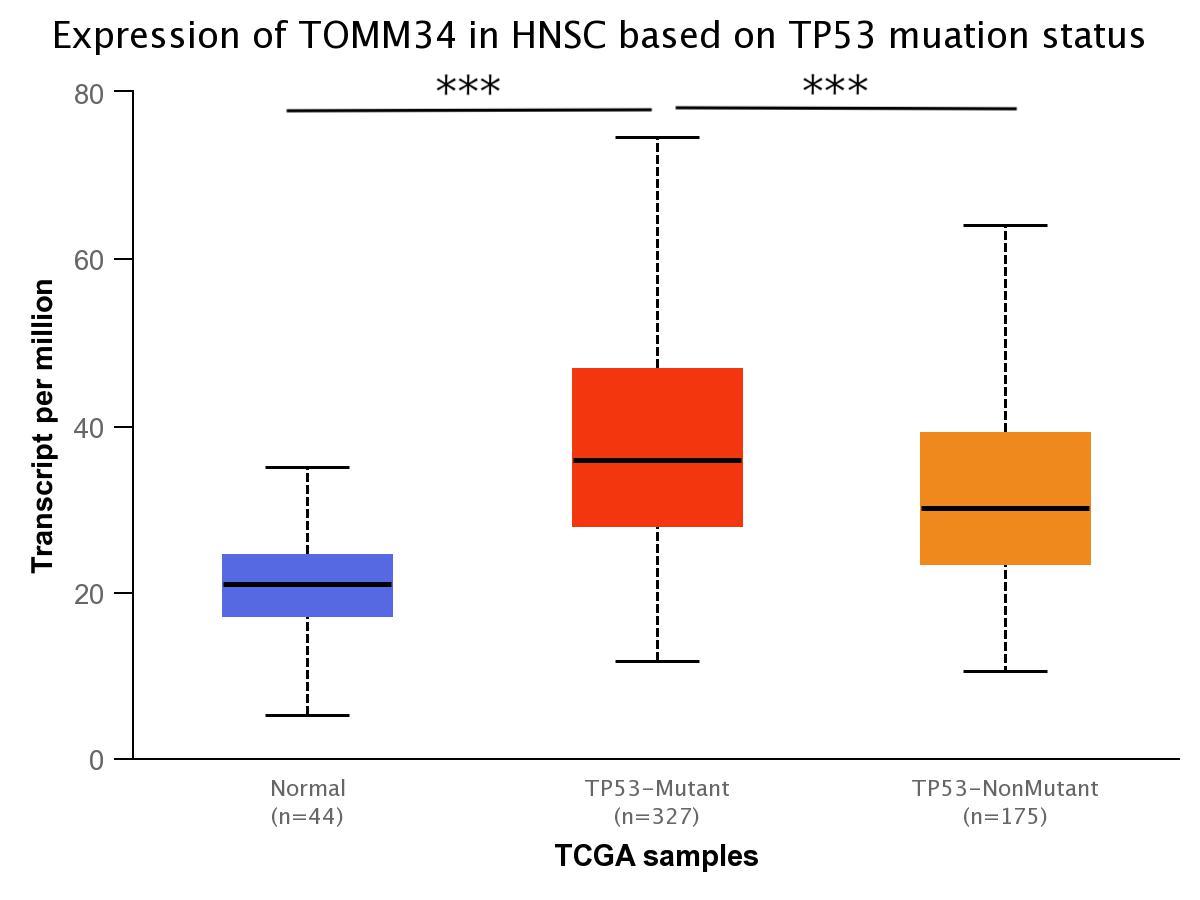

Supplement: Supplementary file 1 [file Image1.jpg]
